# Ocular bacterial infections at Quiha Ophthalmic Hospital, Northern Ethiopia: an evaluation according to the risk factors and the antimicrobial susceptibility of bacterial isolates

**DOI:** 10.1186/s12879-017-2304-1

**Published:** 2017-03-14

**Authors:** Mebrahtu Teweldemedhin, Muthupandian Saravanan, Araya Gebreyesus, Dawit Gebreegziabiher

**Affiliations:** 1 Department of Medical Microbiology and Immunology, Institute of Biomedical Sciences, College of Health Sciences, Mekelle University, 1871, Mekelle, Ethiopia; 2 Unit of Biomedical Science, School of Medicine, College of Health Sciences, Aksum University, Aksum, Tigray Ethiopia

**Keywords:** Bacterial occurrence, Ocular infection, Risk factor, Antimicrobial susceptibility, Multidrug resistance

## Abstract

**Background:**

External and intraocular infections can lead to visual impairments, which is a major public health problem. Bacteria are the most frequent pathogens affecting ocular structures; the increasing rate of antimicrobial drug resistance is a worldwide concern. The aim of this study was to determine the occurrence of bacteria in ocular infections, their antimicrobial susceptibility patterns, and risk factors in bacterial ocular infection.

**Methods:**

A hospital based cross-sectional study was conducted from September 2015 to December 2015 at Quiha Ophthalmic Hospital, Tigray, northern Ethiopia. Ocular specimens from blepharitis, blepharoconjunctivitis, conjunctivitis, keratitis, endophthalmitis, periorbital cellulitis and dacrocystitis were collected from 270 individuals with suspected ocular infection. Data on sociodemographic and risk factors were also collected using a structured questionnaire. Data analysis was performed using SPSS version 21 and 0.05 with a corresponding 95% confidence interval (CI) was considered statistically significant.

**Results:**

Among 270 study subjects, 180 (66.7%) were culture positive for different bacterial isolates. The predominant bacterial isolates were *Staphylococcus aureus* (40, 22.2%), coagulase negative staphylococci (31, 17.2%) and *Pseudomonas aeruginosa* (21, 11.7%). Ocular surface disease, ocular trauma, hospitalization and cosmetic application practices were significantly associated with the occurrence of bacterial infection. Concerning antimicrobial susceptibility, most isolates were susceptible to amikacin (137, 93.2%), gentamicin (131, 89.1%) and ciprofloxacin (141, 89.2%). Overall, 40 (22.5%), 34 (19.1%) and 62 (34.8%) isolates were resistant to one, two, and three or more antimicrobials, respectively.

**Conclusion:**

Bacteria were isolated from the majority of the study subjects. More than half of the bacterial isolates were resistant at least to one drug and a significant rate of multidrug resistance was detected. Therefore, identification of the etiologic agent and antimicrobial susceptibility testing should be practiced to select the appropriate antimicrobial agent to treat eye infections and prevent the emergence of drug resistant bacteria.

## Background

The eye, a functionally and structurally complex organ, experiences a variety of bacterial, viral, fungal and parasitic infections [1–3]. Bacterial infections (Both Gram-positive and Gram-negative) contribute to 32 to 74% of ocular infections globally [4–10]. *Staphylococci* are the leading ocular isolates worldwide among the Gram-positive bacteria, [9, 11–13]; while *Pseudomonas aeruginosa*, *Klebsiella pneumoniae* and *Escherichia coli* are the major Gram-negative bacteria isolated from ocular infections [3, 14, 15].

Bacteria are the most common agents causing external ocular infections, including blepharitis, keratitis, dacryocystitis, and orbital cellulitis. Bacteria are responsible for 70–80% of conjunctival morbidity which poses a huge socioeconomic burdens to the general public [16–19]. Blepharitis, an inflammation of the eyelid, can cause complete loss of the eyelashes if not diagnosed early [20]. Bacterial keratitis is the leading cause of corneal blindness [18, 21, 22].

External ocular infections may remain localized, or progress to adjacent tissues. For instance, external and internal hordeolum can result from the spread of eyelid infection to the respective glands [23]. Dacryocystitis, inflammation of the nasolacrimal duct, is the result of nasolacrimal duct obstruction. The accumulation of fluid and edema in the eye secondary to dacryocystitis, is a potential danger to ocular tissues such as the cornea and conjunctiva [24, 25]. External ocular infections that result from systemic spread, surgery or are secondary to ocular trauma, can lead to sight threatening intraocular infections such as endophthalmitis [1, 22, 26, 27]. Left untreated, bacterial ocular infections, bacterial products and vigorous inflammation following infection, can irreversibly damage ocular structures [28, 29]. Inflammation and scarring, once present, may not be easily resolved and can result in visual impairment or permanent loss of vision [30, 31].

Additionally, bacterial ocular infections have been complicated by multidrug resistance;, a problem that is intensifying over time [32, 33]. This poses a challenge in the clinical management of bacterial ocular infections [34–36].

Eye infections are remarkably common in northern Ethiopia; however, no studies have been conducted on this topic in this region. Similarly, the antimicrobial susceptibility patterns of infecting bacteria in this region are not known. Therefore, we sought to determine the ocular bacterial infection, the risk factors and the antimicrobial susceptibility of bacterial isolates in ocular infections at Quiha Ophthalmic Hospital, Tigray, northern Ethiopia.

## Methods

### Study design, data and specimens collection

A hospital based cross-sectional study was conducted from September-December 2015 in Quiha Ophthalmic Hospital, Quiha, Tigray, Ethiopia. A total of 270 patients who presented with suspected ocular infection were consecutively included in the study. Sociodemographic, clinical and risk-factor related data were collected using a pretested structured questionnaire using interviewer-administered questionnaires. Study subjects were examined using a slit-lamp biomicroscope to screen for the presence of ocular infection. Ocular specimens were collected from periorbital cellulitis (*N* = 8), dacryocystitis (*N* = 13), blepharitis (*N* = 26), blepharoconjunctivitis (*N* = 16), conjunctivitis (*N* = 123), keratitis (*N* = 57) and endophthalmitis (*N* = 27) diagnoses. An ophthalmic surgeon collected specimens from all patients except those patients who had endophthalmitis who had their specimemens collected by an ophthalmologist [37–39].

### Specimen transportation

The respective specimens were then transferred into 2 ml of brain-heart infusion broth (Oxoid, Hampshire, UK). Tubes were tightly capped, gently mixed, labeled and placed in cold chain. Finally, samples were transported to the Microbiology Laboratory, Mekelle University, for microbiological analysis [38, 39].

### Cultivation and identification of isolates

The broth was gently mixed to homogeneity; 100 μl of inoculum was then dispensed and streaked onto blood agar, chocolate agar, MacConkey agar, Mannitol salt agar and modified Thayer-Martin agar (MTM) (Oxoid, Hampshire, UK). To maintain the presence of CO_2_, chocolate agar and MTM agar plates were placed in candle jars. All media were incubated overnight at 37 °C. After 24 h of incubation, each plate was inspected for any growth and negative plates were incubated for an additional 24 h. For eyelid and conjunctival swabs, culture positivity was determined based on a threshold criteria. Corneal specimens were considered as positive if there was a confluent growth at the site of inoculation [39]. All bacterial isolates were identified using standard clinical laboratory methods [40, 41].

### Antimicrobial susceptibility testing

Antimicrobial susceptibility testing was performed using the standard Kirby-Bauer disk diffusion method [41]. For fastidious organisms, Muller-Hinton agar with 5% sheep blood was used and incubated in 5% CO_2_; GC agar and *Haemophilus* Test Medium were used for *Neisseria gonorrhoeae* and *Haemophilus influenzae*, respectively [42]. The following antibiotics were used: trimethoprim-sulfamethoxazole (SXT; 1.25/23.75 μg), erythromycin (15 μg), clarithromycin (15 μg), chloramphenicol (30 μg), clindamycin (2 μg), tetracycline (30 μg), doxycycline (30 μg), amikacin (30 μg), gentamicin (10 μg), ciprofloxacin (5 μg), ceftriaxone (30 μg) and cefoxitin (30 μg) (HIMEDIA). The zones of inhibition of the antimicrobial agents were measured using calipers; bacteria were described as susceptible or resistant based on CLSI guideline [42]. Multidrug resistance was defined as non-susceptible to ≥1 agent in ≥3 antimicrobial categories according to the definition of Magiorakos and colleagues [43].

### Quality assurance

Standard operational procedures were strictly followed from the pre-analytical to the post-analytical phase. The questionnaire was pretested on 16 patients with ocular infection. Data collectors were trained on data collection procedures. Completed questionnaires were proofread immediately after completion by the research team to clarify any labelling errors or illegibility. Standard Operating procedures were followed during Specimen collection, handling, transportation, microbiological analysis and interpretation of results. Reagents, media and antimicrobial disks were checked for the expiry date, damage and storage problems. Media sterility was strictly monitored by random sampling and incubation to test for any growth. In addition, reference strains of *Staphylococcus aureus* ATCC25923, *E. coli* ATCC25922 and *P. aeruginosa* ATCC27853 were used to monitor the quality of this work [42].

### Statistical analysis

Data was entered into SPSS version 21, and descriptive statistics, bivariate and multivariate logistic regression analyses were performed. Bivariate logistic regression was employed to look for association between the outcome variable and independent variables. Variables with *P*-values <0.05 were reanalyzed in multivariate logistic regression to identify risk factors for bacterial occurrence. The corresponding *P*-value <0.05 and the confidence interval (CI) of 95% were considered statistically significant.

## Results

### Sociodemographic characteristics

A total of 270 study subjects with clinically diagnosed ocular infections were included in the study, of whom 151 (55.9%) females, 119 (44.1%) were males and 158 (58.5%) were rural dwellers (Table 1). The mean (SD) age of the study subjects was 37.8 (22.9) years.

**Table 1:** Sociodemographic characteristics of the study subjects (*N* = 270)

Characteristic	Number *N* = 270	Percentage (%)
Gender		
Male	119	44.1
Female	151	55.9
Age (years) (mean (SD) = 37.8 22.9)		
Less than 16		
16–30	58	21.5
31–45	49	18.1
46–60	64	23.7
Greater than 60	51	18.9
	48	17.8
Residence		
Rural	158	58.5
Urban	112	41.5
Occupation		
Preschool	30	11.1
Student	40	14.8
Housewife	43	15.9
Farmer	91	33.7
Governmental employee	34	12.6
Self-employed	32	11.9
Total	270	100
Educational level		
Preschool	30	11.1
1–8 grades	66	24.1
9–12 grades	34	12.6
College and above	18	6.7
No formal education	122	45.2
Total	270	100

### Bacterial profile and clinical features

Conjunctivitis (123 cases of 270, 45.6%) was the leading clinical presentation, followed by keratitis (57, 21.1%), endophthalmitis (27, 10.0%), blepharitis (26, 9.6%), blepharoconjunctivitis (16, 5.9%), dacryocystitis (13, 4.8%), and periorbital cellulitis (8, 3.0%) (Table 2). Of the total 270 patients, 180 (66.7%) were culture positive for bacterial isolates. Single and mixed bacterial infection patients were seen in 174 (96.7%) and 6 (3.3%) cases, respectively (Table 2). The mixed isolates were detected from three patients with conjunctivitis, one patient with periorbital cellulitis, one with keratitis and one with endophthalmitis diagnosis.

**Table 2:** The distribution of culture positive samples in ocular infection

Clinical diagnosis	Bacterial Culture positive		Total, *N* (%)
	No (%)	Yes (%)	
Periorbital cellulitis	1 (12.5)	7 (87.5)	8 (100)
Dacryocystitis	1 (7.7)	12 (92.3)	13 (100)
Blepharitis	12 (46.2)	14 (53.8)	26 (100)
Blepharoconjunctivitis	10 (62.5)	6 (37.5)	16 (100)
Conjunctivitis	48 (39)	75 (61)	123 (100)
Keratitis	11 (19.3)	46 (80.7)	57 (100)
Endophthalmitis	7 (25.9)	20 (74.1)	27 (100)

Bacterial infection was significantly higher in patients with dacryocystitis 92.3% (COR = 20, 95% CI: 2.0–195, *P* = 0.01), periorbital cellulitis 87.5% (COR = 11.7, 95% CI: 1.1–183.8, *P* = 0.03), keratitis 80.7% (COR = 6.9, 95% CI: 2.0–23.3, *P* < 0.01) and endophthalmitis 74.1% (COR = 4.7, 95% CI: 1.2–17.9, *P* = 0.02) than for the other diagnoses (Table 2).

### Identification of bacterial isolates

In this study, 113 (60.7%) bacterial isolates were Gram-positive, and 87 (39.3%) were Gram-negative. Overall, the dominant bacterial isolates were *S. aureus* (40, 21.5%) followed by coagulase-negative staphylococci (CoNS; 31, 16.7%), *P. aeruginosa* (21, 11.3%) and *E. coli* (15 8%) (Table 3).

**Table 3:** The distribution of bacteria according to clinical diagnosis

Type of bacterial isolate, *N* (%)	Clinical diagnosis							Total *N* (%)
	P’orbital cellulitis^a^	Dacryocystitis	Blepharitis	Blepharoconjunctivitis	Conjunctivitis^b^	Keratitis^c^	Endophthalmitis^d^	
*S. aureus*	4 (50)	3 (25)	5 (38.5)	2 (33.3)	17 (21.5)	7 (14.9)	2 (9.5)	40 (21.5)
CoNS^e^	0	0	3 (23.1)	1 (16.7)	13 (16.5)	8 (17)	6 (28.6)	31 (16.7)
*S. pyogenes*	1 (12.5)	3 (25)	0	0	1 (1.3)	0	0	5 (2.7)
*S. pneumoniae*	2 (25)	2 (16.7)	0	1 (16.7)	3 (3.8)	0	0	8 (4.3)
Viridans streptococci	0	0	0	0	6 (7.6)	1 (2.1)	0	7 (3.8)
*Enterococcus* spp*.*	0	0	0	0	6 (7.6)	2 (4.3)	0	8 (4.3)
*Moraxella* spp*.*	1 (12.5)	0	0	0	0	1 (2.1)	0	2 (1.1)
*N. gonorrhoeae*	0	0	0	0	1 (1.3)	0	0	1 (0.5)
*H. influenzae*	0	0	0	0	2 (2.5)	0	0	2 (1.1)
*P. aeruginosa*	0	0	1 (7.7)	0	2 (2.5)	11 (23.4)	7 (33.3)	21 (11.3)
*K. pneumoniae*	0	1 (8.3)	1 (7.7)	0	2 (2.5)	1 (2.1)	2 (9.5)	7 (3.8)
*Klebsiella* spp*.*	0	1 (8.3)	0	0	2 (2.5)	2 (4.3)	0	5 (2.7)
*E. coli*	0	1 (8.3)	1 (7.7)	1 (16.7)	7 (8.9)	4 (8.5)	1 (4.8)	15 (8.1)
*Enterobacter* spp*.*	0	1 (8.3)	1 (7.7)	0	5 (6.3)	3 (6.4)	0	10 (5.4)
*Citrobacter freundii*	0	0	0	0	2 (2.5)	2 (4.3)	0	4 (2.2)
*S. marcescens*	0	0	0	1 (16.7)	3 (3.8)	1 (2.1)	0	5 (2.7)
*Aeromonas* spp*.*	0	0	0	0	1 (1.3)	1 (2.1)	0	2 (1.1)
*P. shigelloides*	0	0	0	0	1 (1.3)	0	0	1 (0.5)
*Proteus* spp*.*	0	0	0	0	2 (2.5)	1 (2.1)	0	3 (1.6)
*Acinetobacter* spp*.*	0	0	1 (7.7)	0	1 (1.3)	2 (4.3)	1 (4.8)	5 (2.7)
GPB^f^	0	0	0	0	2 (2.5)	0	2 (9.5)	4 (2.2)
Total	8 (100)	12 (100)	13 (100)	6 (100)	79 (100)	47 (100)	21 (100)	186 (100)

*Notes*: ^a^mixed isolate in one case: *S. pneumoniae* + *H. influenzae;* ^b^mixed isolates in three cases: *E. coli* + *P. aeruginosa*; *E. coli* + *S. aureus & S. marcescens + S. aureus ;* ^c^mixed isolate in one case: *P. aeruginosa* + *S. aureus ;* ^d^mixed isolate in one case: *E. coli* + CoNS; ^e^ *CoNS* Coagulase negative staphylococci; ^f^ *GPB* Gram-positive bacilli, P’orbital cellulitis = Periorbital cellulitis

### Risk factors for bacterial occurrence in ocular infections

In this study, 11 independent variables were considered during the bivariate analysis of risk factors for bacterial infection. In multivariate analysis, ocular surface disease (AOR = 13.6, 95% CI: 3.8 49.3, *P* < 0.01), ocular trauma (AOR = 4.2, 95% CI: 1.4–13, *P* = 0.012), hospitalization (AOR = 3.3, 95% CI: 1.03–12.5, *P* = 0.04), and cosmetic application practices (AOR = 4.7, 95% CI: 1.6–13.9 *P* < 0.01) were significantly associated with bacterial occurrence (Table 4). Though not statistically significant, bacterial occurrence was higher in housewives 32 (74.4%) (COR = 2.3, 95% CI: 0.6–9.0, *P* = 0.06) and in those subjects with no formal education 90 (73.8%) (COR = 1.8, 95% CI: 0.6–5.0, *P* = 0.2) (Table 5).

**Table 4:** Multivariate logistic regression analysis of factors associated with ocular bacterial infections

Variable	Bacterial occurrence, N (%)		Total, *N* (%)	COR^a^ (95% CI^b^), *P*-value	AOR^c^ (95% CI), *P*-value
	No	Yes			
History of ocular surface disease					
Yes	10 (11.0)	81 (89.0)	91 (100)	6.5 (3.2–13.4), <0.01	13.6 (3.8–49), <0.01
No	80 (44.7)	99 (53.3)	179 (100)	1	1
History of ocular trauma					
Yes	14 (16.7)	70 (83.3)	84 (100)	3.4 (1.8–6.5), <0.01	4.2 (1.4–13), 0.012
No	76 (40.9)	110 (59.1)	186 (100)	1	1
History of hospitalization					
Yes	9 (13.4)	58 (86.6)	67 (100)	4.3 (2–9), <0.01	3.3 (1.03–12.5), 0.04
No	81 (39.9)	122 (60.1)	203 (100)	1	1
Cosmetic application practices					
Yes	7 (12.1)	51 (87.9)	58 (100)	5.3 (2.2–12.8), <0.01	4.7 (1.6–13.9), <0.01
No	39 (41.9)	54 (58.1)	93 (100)	1	1

*Notes:* ^*a*^ *COR* Crude odds ratio; ^b^ *CI* Confidence interval; ^c^ *AOR* Adjusted odds ratio

**Table 5:** Bivariate logistic regression analysis of factors associated with ocular bacterial infections

Variable	Bacterial occurrence *N* (%)		Total *N* (%)	COR^a^	95% CI^b^	*P*-value
	No	Yes				
Gender						
Male	41 (34.5)	78 (65.5)	119 (100)	0.9	0.5–1.5	0.7
Female	49 (32.5)	102 (67.5)	151 (100)	1		
Age						
15	28 (48.3)	30 (51.7)	58 (100)	1		
16–30	15 (30.6)	34 (69.4)	49 (100)	2.1	0.9–4.6	0.06
31–45	17 (26.6)	47 (73.4)	64 (100)	2.6	1.2–5.5	0.01
46–60	14 (27.5)	37 (72.5)	51 (100)	2.5	1.1–5.5	0.02
61	16 (33.3)	32 (66.7)	48 (100)	1.8	0.8–4.1	0.1
Residence						
Rural	48 (30.4)	110 (69.6)	158 (100)	0.7	0.4–1.2	0.22
Urban	42 (37.5)	70 (39.1)	112 (100)	1		
Occupation						
Preschool	13 (43.3)	17 (56.7)	30 (100)	1.0	0.4–2.7	0.8
Student	18 (45.0)	22 (55.0)	40 (100)	1		
Housewife	11 (25.6)	32 (74.4)	43 (100)	2.3	0.9–6.0	0.06
Farmer	25 (27.5)	66 (72.5)	91 (100)	2.1	0.9–4.6	0.051
Government employee	13 (38.2)	21 (61.8)	34 (100)	1.3	0.5–3.3	0.2
Self-employed	10 (31.3)	22 (68.8)	32 (100)	1.8	0.6–4.7	0.2
Educational level						
Preschool	13 (43.3)	17 (56.7)	30 (100)	0.8	0.25–2.7	0.7
1–8	28 (42.4)	38 (57.6)	66 (100)	0.9	0.3–2.5	0.8
9–12	10 (29.4)	24 (70.6)	34 (100)	1.5	0.7–4.2	0.2
College and above	7 (38.9)	11 (61.1)	18 (100)	1		
No formal education	32 (26.2)	90 (73.8)	122 (100)	1.8	0.6–5.0	0.2
History of ocular surface disease						
Yes	10 (11.0)	81 (89.0)	91 (100)	6.5	3.2–13.4	**<0.01**
No	80 (44.7)	99 (53.3)	179 (100)	1		
History of Ocular surgery						
Yes	14 (24.6)	43 (75.4)	57 (100)	1.7	0.8–3.3	0.11
No	76 (35.7)	137 (64.3)	213 (100)	1		
History of Ocular trauma						
Yes	14 (16.7)	70 (83.3)	84 (100)	3.4	1.8–6.5	**<0.01**
No	76 (40.9)	110 (59.1)	186 (100)	1		
History of Hospitalization						
Yes	9 (13.4)	58 (86.6)	67 (100)	4.3	2–9	**<0.01**
No	81 (39.9)	122 (60.1)	203 (100)	1		
Contact lens use						
Yes	4 (30.8)	9 (69.2)	13 (100)	1.1	0.3–3.7	0.8
No	86 (33.5)	171 (66.5)	257 (100)	1		
Cosmetic application practices						
Yes	7 (12.1)	51 (87.9)	58 (100)	5.3	2.2–12.8	**<0.01**
No	39 (41.9)	54 (58.1)	93 (100)	1		

*Notes*: ^a^ *COR* Crude odds ratio; ^b^ *CI* Confidence interval; *P*-values in bold are statistically significant

### Antimicrobial susceptibility pattern of bacterial isolates

In this study, the majority of bacterial isolate were susceptible to ciprofloxacin (141, 89.2%), amikacin (137, 93.2%), gentamicin (131, 89.1%), chloramphenicol (106, 70.2%), and doxycycline (100, 71.9%). However, bacterial isolates were less susceptible to tetracycline (79, 51.3%) and SXT (66, 48.7%). Among the Gram-negative bacteria, 47 bacterial isolates (83%) were susceptible to ceftriaxone. In addition, 79 (70.7%) of the Gram-positive isolates were susceptible to erythromycin. All isolates of *S. pyogenes*, *S. pneumoniae*, viridans streptococci and *H. influenzae* were susceptible to clarithromycin. Methicillin-resistance was observed in 7 (17.5%) *S. aureus* and 14 (45.2%) CoNS isolates (Table 6).

**Table 6:** Antimicrobial susceptibility of bacteria isolated from ocular infections

Bacterial isolate (N)	S/R N (%)	Antibiotics tested											
		CN	AK	C	CIP	TC	DOX	SXT	CRO	DA	E	CH	MET
*S. aureus* (40)	R S	5 (12.5) 35 (87.5)	1 (2.5) 39 (97.5)	11 (27.5) 29 (72.5)	5 (12.5) 35 (87.5)	18 (45) 22 (55)	11 (27.5) 29 (72.5)	17 (42.5) 23 (57.5)	NT	9 (22.5) 31 (77.5)	8 (20) 32 (80)	NT	7 (17.5) 33 (82.5)
CoNS^a^ (31)	R S	2 (6.5) 29 (93.5)	1 (3.2) 30 (96.8)	9 (29) 22 (71)	3 (9.7) 28 (90.3)	18 (58.1) 13 (41.9)	7 (22.6) 24 (77.4)	21 (67.7) 10 (32.3)	NT	12 (38.7) 19 (61.3)	9 (29) 22 (71)	NT	14 (45.2) 17 (54.8)
*S. pyogenes* (5)	R S	NT	NT	2 (40) 3 (60)	NT	1 (20) 4 (80)	NT	NT	NT	0 5 (100)	0 5 (100)	0 5 (100)	NT
*S. pneumoniae* (8)	R S	NT	NT	2 (25) 6 (75)	NT	4 (50) 4 (50)	2 (25) 6 (75)	3 (37.5) 5 (62.5)	NT	1 (12.5) 7 (87.5)	1 (12.5) 7 (87.5)	0 8 (100)	NT
Viridans streptococci (7)	R S	NT	NT	3 (42.9) 4 (57.1)	NT	2 (28.6) 5 (71.4)	NT	NT	0 7 (100)	NT	1 (14.3) 6 (85.7)	0 7 (100)	NT
*Enterococcus* spp*.* (8)	R S	NT	NT	4 (50) 4 (50)	1 (12.5) 7 (87.5)	4 (50) 4 (50)	2 (25) 6 (75)	NT	NT	NT	1 (12.5) 7 (87.5)	NT	NT
*N. gonorrhoeae* (1)	R S	NT	NT	NT	0 1 (100)	0 1 (100)	NT	NT	0 1 (100)	NT	NT	NT	NT
*H. influenzae* (2)	R S	NT	NT	0 2 (100)	0 2 (100)	0 2 (100)	NT	1 (50) 1 (50)	NT	NT	NT	0 2 (100)	NT
*P. aeruginosa* (21)	R S	1 (4.8) 20 (95.2)	1 (4.8) 20 (95.2)	NT	4 (19) 17 (81)	NT	NT	NT	NT	NT	NT	NT	NT
*K. pneumoniae* (7)	R S	2 (28.6) 5 (71.4)	1 (14.3) 6 (85.7)	3 (42.9) 4 (57.1)	1 (14.3) 6 (85.7)	5 (71.4) 2 (28.6)	2 (28.6) 5 (71.4)	4 (57.1) 3 (42.9)	3 (42.9) 4 (57.1)	NT	NT	NT	NT
Other *Klebsiella spp.* (5)	R S	0 5 (100)	1 (20) 4 (80)	2 (40) 3 (60)	1 (20) 4 (80)	2 (40) 3 (60)	1 (20) 4 (80)	4 (80) 1 (20)	0 5 (100)	NT	NT	NT	NT
*E. coli* (15)	R S	3 (20) 12 (80)	2 (13.3) 13 (86.7)	4 (26.7) 11 (73.3)	1 (6.7) 14 (93.3)	8 (53.3) 7 (46.7)	3 (20) 12 (80)	8 (53.3) 7 (46.7)	3 (20) 12 (80)	NT	NT	NT	NT
*Enterobacter* spp*.* (10)	R S	1 (10) 9 (90)	1 (10) 9 (90)	1 (10) 9 (90)	0 10 (100)	6 (60) 4 (40)	5 (50) 5 (50)	4 (40) 6 (60)	2 (20) 8 (80)	NT	NT	NT	NT
*Citrobacter freundii* (4)	R S	0 4 (100)	0 4 (100)	2 (50) 2 (50)	1 (25) 3 (75)	3 (75) 1 (25)	2 (50) 2 (50)	1 (25) 3 (75)	0 4 (100)	NT	NT	NT	NT
*Acinetobacter* spp*.* (5)	R S	1 (20) 4 (80)	1 (20) 4 (80)	NT	0 5 (100)	3 (60) 2 (40)	3 (60) 2 (40)	4 (80) 1 (20)	0 5 (100)	NT	NT	NT	NT
*S. marcescens* (5)	R S	1 (20) 4 (80)	1 (20) 4 (80)	2 (40) 3 (60)	0 5 (100)	1 (20) 4 (80)	1 (20) 4 (80)	2 (40) 3 (60)	1 (20) 4 (80)	NT	NT	NT	NT
*Proteus* spp*.* (3)	R S	0 3 (100)	0 3 (100)	0 3 (100)	0 3 (100)	NT	NT	1 (33.3) 2 (66.7)	0 3 (100)	NT	NT	NT	NT
*P. shigelloides* (1)	R S	0 1 (100)	0 1 (100)	0 1 (100)	0 1 (100)	0 1 (100)	0 1 (100)	0 1 (100)	0 1 (100)	NT	NT	NT	NT
Total, N (%)	R S	16 (10.9) 131 (89.1)	10 (6.8) 137 (93.2)	45 (29.8) 106 (70.2)	17 (10.8) 141 (89.2)	75 (48.7) 79 (51.3)	39 (28.1) 100 (71.9)	70 (51.3) 66 (48.7)	9 (14.3) 54 (85.7)	22 (26.2) 62 (73.8)	20 (20.2) 79 (79.8)	0 22 (100)	21 (29.6) 50 (70.4)

*Notes*: *S* Susceptible, *R* Resistant, *CN* Gentamicin, *AK* Amikacin, *C* Chloramphenicol, *CIP* Ciprofloxacin, *TC* Tetracycline, *DOX* Doxycycline, *SXT* Trimethoprim-sulfamethoxazole, *CRO* Ceftriaxone, *DA* Clindamycin, *E* Erythromycin, *CH* Clarithromycin, *MET* Methicillin, *NT* Not tested; ^a^ *CoNS* Coagulase negative staphylococci

Overall, 40 (22.5%) bacterial isolates were resistant to at one antimicrobial agent; 96 (53.9%) were resistant to ≥2 antimicrobials (Table 7). A standard definition of MDR was applied for isolates of *S. aureus*, members of the Enterobacteriaceae and *Acinetobacter* spp. [43], by which 18 (45%) *S. aureus*, 17 (31.5%) isolates of Enterobacteriaceae, and one *Acinetobacter* isolate were found to be MDR. Among Enterobacteriaceae, *Klebsiella* spp. (6, 50%) and *E. coli* (7, 46.7%) were the commonest MDR isolates (Table 8).

**Table 7:** Level of antimicrobial resistance in bacteria isolated from ocular infections

Type of bacterial isolate		Level of resistance *N* (%)					Total
		R0	R1	R2	R3	R4	
Gram-positive	*S. aureus*	7 (17.5)	8 (20)	6 (15)	7 (17.5)	12 (30)	40 (100)
	CoNS^a^	4 (12.9)	6 (19.4)	5 (16.1)	0	16 (51.6)	31 (100)
	*S. pyogenes*	3 (60)	2 (40)	0	0	0	5 (100)
	*S. pneumoniae*	1 (12.5)	2 (25)	4 (50)	0	1 (12.5)	8 (100)
	Viridans streptococci	2 (28.6)	2 (28.6)	2 (28.6)	1 (14.3)	0	7 (100)
	*Enterococcus* spp.	2 (25)	1 (12.5)	4 (50)	1 (12.5)	0	8 (100)
	Total	19 (19.2)	21 (21.2)	21 (21.2)	9 (9)	29 (29.3)	99 (100)
Gram-negative	*N. gonorrhoeae*	1 (100)	0	0	0	0	1 (100)
	*H. influenzae*	1 (50)	1 (50)	0	0	0	2 (100)
	*P. aeruginosa*	10 (47.6)	9 (42.9)	2 (9.5)	0	0	21 (100)
	*K. pneumoniae*	0	0	1 (14.3)	5 (71.4)	1 (14.3)	7 (100)
	Other *Klebsiella* spp.	0	2 (40)	1 (20)	1 (20)	1 (20)	5 (100)
	*E. coli*	5 (33.3)	2 (13.3)	1 (6.7)	1 (6.7)	6 (40)	15 (100)
	*Enterobacter* spp.	2 (20)	2 (20)	3 (30)	1 (10)	2 (20)	10 (100)
	*Citrobacter freundii*	1 (25)	0	1 (25)	1 (25)	1 (25)	4 (100)
	*S. marcescens*	2 (40)	1 (20)	0	1 (20)	1 (20)	5 (100)
	*P. shigelloides*	1 (100)	0	0	0	0	1 (100)
	*Proteus* spp.	0	0	2 (66.7)	1 (33.3)	0	3 (100)
	*Acinetobacter* spp.	0	2 (40)	1 (20)	1 (20)	1 (20)	5 (100)
	Total	23 (29.1)	19 (24)	13 (16.5)	11 (13.9)	13 (16.5)	79 (100)
Grand Total		42 (23.6)	40 (22.5)	34 (19.1)	20 (11)	42 (23.5)	178 (100)

*Notes*: *R0* Susceptible to all tested antimicrobials, *R1* Resistant to one antimicrobial, *R2* Resistant to two antimicrobials, *R3* Resistant to three antimicrobials, *R4* Resistant to four or more antimicrobials; ^a^ *CoNS* Coagulase negative staphylococci

**Table 8:** Multidrug resistance patterns of bacterial isolates from ocular infections

Type of bacterial isolate	Total number of isolates	MDR^a^ isolates, *N* (%)
*S. aureus*	40	18 (45)
*K. pneumoniae*	7	4 (57.1)
Other *Klebsiella* spp.	5	2 (40)
*E. coli*	15	7 (46.7)
*Enterobacter* spp.	10	2 (20)
*Citrobacter freundii*	4	1 (25)
*Serratia marcescens*	5	1 (20)
*P. shigelloides*	1	0
*Proteus* spp.	3	0
*Acinetobacter* spp*.*	5	1 (20)
Total	95	36 (37.9)

*Notes*: ^a^ *MDR* Multi-drug resistant: non-susceptible to 1 agent in 3 antimicrobial categories [43]. Based on this definition, the following antimicrobial categories were considered to determine whether the given isolate is MDR: *S. aureus*: aminoglycosides, methicillin, SXT, clindamycin, chloramphenicol, macrolides (erythromycin), tetracyclines and fluoroquinolones (ciprofloxacin). Enterobacteriaceae: aminoglycosides, ceftriaxone, fluoroquinolones (ciprofloxacin), SXT, chloramphenicol and tetracyclines. *Acinetobacter* spp.: aminoglycosides, fluoroquinolones (ciprofloxacin), ceftriaxone, SXT and tetracyclines

## Discussion

Our assessment revealed that a high proportion of ocular infections (66.7%) were due to bacterial infections elsewhere in Ethiopia, lower proportions have been observed (48.8–60.8%) [10, 11, 44]; however, higher proportions (74.7%) have been observed in Jimma, Ethiopia [6]. This variation could be attributed to the inclusion of only exra-ocular infection in other studies. Our observations were also higher than that seen in India (34.5%) [3], Japan (32.2%) [4], and Iran (37.5%) [45]; sociodemographic, geographical or climatic differences for the patient populations could partially explain this [38]. Similar to findings from India [46], Iran [9], and other studies in Ethiopia [6, 10, 11, 44], Gram-positive bacteria contributed to majority (60.7%) of the total bacterial isolates in our study. Overall, the predominant bacterial isolate was *S. aureus* (21.5%), as has been reported in Jimma (28.4%) [6], Gondar (21%) [10], Nigeria (23.7%) [23], and India (26.6%) [47]. Staphylococcal isolates were predominant among patients with conjunctivitis, blepharitis and blepharoconjunctivitis diagnoses, similar to that in patients from Jimma [6], Nigeria [23], and Columbia [48]. Conversely,, CoNS isolates were more frequent in keratitis (17%) and endophthalmitis (28.6%) diagnoses, as has been reported in Uganda [49], Mexico [50], Australia [8], Pakistan [51] and India [52].

As compared to published literature from other areas in Ethiopia, Nigeria, UK, Australia and India, most streptococcal isolates were from conjunctivitis diagnoses, with some isolates from blepharoconjunctivitis and keratitis. In the literature, Staphylococci and streptococci are known to be the main agents of post-traumatic periorbital cellulitis [54]; in our study, *S. aureus* (50%), *S. pneumoniae* (12.5%) and *S. pyogenes* (12.5%) were the main Gram-positive isolates from periorbital cellulitis. As has been seen in Gondar [19], Borumeda [44], and India [47], *S. pneumoniae* and *S. pyogenes* were the predominant species among patients with dacryocystitis.

The most common isolates among the Gram-negative bacteria were *P. aeruginosa* (11.3%), *E. coli* (8.1%) and *Klebsiella* spp. (6.4%) as has been seen in Hawassa; however, the proportions of *P. aeruginosa* (4.9%) and *E. coli* (4.9%) observed in Hawassa were lower than in our study [23]. This may be due to the additional isolates from endophthalmitis diagnoses we identified.

*P. aeruginosa* was the predominant isolate in keratitis (23.4% of diagnoses) similar to findings reported in Nigeria (23.8%) [23], the UK (24.3%) [7], Australia (21%) [8], and Iran (24.7%) [15]. Pseudomonal keratitis is a progressive infection with large infiltrate and scarring [22, 53]; this therefore means majority of patients in our population are at risk of blindness.

Our assessment illustrated that the presence of ocular surface disease, ocular trauma, hospitalization and cosmetic application practices were significantly associated with the occurrence of bacterial infection. Risk factors for bacterial ocular infections that have been described include a history of hospital stay in Columbia and Portugal [48, 55], ocular trauma in Australia, Florida, Iran, China and Mexicao [1, 8, 15, 22, 50] and ocular disease in Florida and Colombia [22, 48] Cosmetic application practices were considered only for female participants. Limited information exists on the relationship between cosmetic application practices and bacterial ocular infections.

Almost one quarter of the bacterial isolates in were susceptible to all of the tested antimicrobials. Resistance to two or more antimicrobials was seen in 53.9% of the isolates, which is lower than that reported in other regions in Ethiopia (69.9–87.1%) [10, 11]. *S. pneumoniae* (87.5%), CoNS (87%) and *S. aureus* (82.5%) were the most resistant among Gram-positive isolates. *Acinetobacter* spp. (100%), *Klebsiella* spp. (100%) and *Enterobacter* spp. (80.0%) were the most resistant among Gram-negative isolates. which is lower than that reported in other studies [56, 57].

Here, relatively, amikacin (93.2%), gentamicin (89.1%) and ciprofloxacin (89.2%) were revealed high efficacy towards Gram-positive and Gram-negative isolates. Studies in Gondar have reported lower susceptibility to gentamicin (54.8%) and ciprofloxacin (74.2%) [10, 19]; differences in the variety of isolates tested may partly explain this. Outside Ethiopia, comparable susceptibility patterns to amikacin, ciprofloxacin and chloramphenicol were reported in Iran [15] and India [47]. However, the gentamicin susceptibility in this study is much higher than that in the study conducted in India (58.6%) [47]; regional variations in antibiotic prescription practices could explain this [38]. The resistance we observed to tetracycline and erythromycin was high, and this paralleled a study from India [3]. High resistance is usually due to the over use and empirical treatment of patients that ultimately leads to the emergence of drug resistant strains [58, 59].

Methicillin resistance was seen in 17.5% of the isolated *S. aureus,* which is in line with a study conducted in India (14%) [60], but lower than the rates in Taiwan (52.8%) [57] and Uganda (31.9%) [49]. In addition, 45.2% of CoNS were methicillin resistant, which was higher than detected in Uganda (27.6%) [49]. To date, other than a study conducted in Gondar, most studies in Ethiopia did not determine methicillin resistance rates for ocular staphylococcal isolates [19]. Our study was not without limitation; we did not isolate anaerobic bacteria or *Chlamydia trachomatis* due to unavailability of the prerequisite facilities. We also did not determine the antimicrobial susceptibility of *Moraxella* species or *Aeromonas* species due to a lack of antimicrobials for agar dilution.

## Conclusions

In conclusion, a high prevalence of bacteria was found in patients with ocular infection. “Ocular surface disease, ocular trauma, hospitalisation and cosmetic application practices were significantly associated with the occurrence of bacterial infection. Overall, *S. aureus*, CoNS, and *P. aeruginosa* were the predominant isolates. The majority of the bacterial isolates were multidrug resistant. Methicillin resistance was higher in CoNS than in S. aureus. *S. pneumoniae*, CoNS of which S. aureus was the most resistant species among Gram-positive isolates; *Klebsiella species* and *E. coli* were most resistant among Gram-negative isolates. Identification of the specific etiologic agent and antimicrobial susceptibility testing should be practiced during the management of ocular infections to reduce the further emergence of multidrug-resistant bacteria”.

## Abbreviations

AOR: Adjusted Odds Ratio
ATCC: American Type Culture Collection
CAMP: Christie, Atkins and Munch-Peterson
CEO: Chief Executive Officer
CI: Confidence interval
CLSI: Clinical and Laboratory Standards Institute
CoNS: Coagulase Negative Staphylococci
COR: Adjusted Odds Ratio
ID: Identification number
MDR: Multi-drug Resistance
MRSA: Methicillin resistant *Staphylococcus aureus*
MRSE: Methicillin Resistant *Staphylococcus epidermidis*
MTM: Modified Thayer-Martin
PCR: Polymerase Chain Reaction
SPSS: Statistical Package for Social Sciences
